# Cycle-dependent sex differences in expression of membrane proteins involved in cerebrospinal fluid secretion at rat choroid plexus

**DOI:** 10.1186/s12868-023-00829-w

**Published:** 2023-11-09

**Authors:** Ida Marchen Egerod Israelsen, Christina Kamp-Jensen, Connar Stanley James Westgate, Bjarne Styrishave, Rigmor H. Jensen, Sajedeh Eftekhari

**Affiliations:** 1 Danish Headache Center, Department of Neurology, Glostrup Research Institute, Rigshospitalet-Glostrup, University of Copenhagen, Nordstjernevej 42, 2600 Glostrup, Denmark; 2 Department of Pharmacy, Faculty of Health and Medical Sciences, University of Copenhagen, Copenhagen, Denmark

**Keywords:** Choroid plexus, Sex-differences, Transporters, Cerebrospinal fluid, Estrous cycle, Hormones, Idiopathic intracranial hypertension

## Abstract

**Background:**

Female sex is a known risk factor of brain disorders with raised intracranial pressure (ICP) and sex hormones have been suggested to alter cerebrospinal fluid (CSF) dynamics, thus impairing ICP regulation in CSF disorders such as idiopathic intracranial hypertension (IIH). The choroid plexus (CP) is the tissue producing CSF and it has been hypothesized that altered hormonal composition could affect the activity of transporters involved in CSF secretion, thus affecting ICP. Therefore, we aimed to investigate if expression of various transporters involved in CSF secretion at CP were different between males and females and between females in different estrous cycle states. Steroid levels in serum was also investigated.

**Methods:**

Female and male rats were used to determine sex-differences in the genes encoding for the transporters Aqp1 and 4, NKCC1, NBCe2, NCBE; carbonic anhydrase enzymes II and III (CA), subunits of the Na^+^/K^+^-ATPase including Atp1a1, Atp1b1 and Fxyd1 at CP. The estrous cycle stage metestrus (MET) and estrous (ES) were determined before euthanasia. Serum and CP were collected and subjected to RT-qPCR analysis and western blots. Serum was used to measure steroid levels using liquid chromatography tandem mass spectrometry (LC–MS/MS).

**Results:**

Significant differences in gene expression and steroid levels between males and ES females were found, while no differences were found between male and MET females. During ES, expression of Aqp1 was lower (p < 0.01) and NKCC1 was higher in females compared to males. CAII was lower while CAIII was higher in ES females (p < 0.0001). Gene expression of Atp1a1 was lower in ES compared to male (p = 0.0008). Several of these choroidal genes were also significantly different in MET compared to females in ES. Differences in gene expression during the estrus cycle were correlated to serum level of steroid hormones. Protein expression of AQP1 (p = 0.008) and CAII (p = 0.035) was reduced in ES females compared to males.

**Conclusions:**

This study demonstrates for the first time that expression at CP is sex-dependent and markedly affected by the estrous cycle in female rats. Further, expression was related to hormone levels in serum. This opens a completely new avenue for steroid regulation of the expression of CSF transporters and the close link to the understanding of CSF disorders such as IIH.

**Supplementary Information:**

The online version contains supplementary material available at 10.1186/s12868-023-00829-w.

## Introduction

Many neurological pathologies are over-represented in females including disorders of cerebrospinal fluid (CSF) dynamics, in particular idiopathic intracranial hypertension (IIH) which is characterized by raised CSF pressure mainly affecting obese women [1, 2]. Approximately 80% of CSF is produced and secreted by the choroid plexus (CP), a unique structure consisting of a single layer of epithelial cells that reside in the cerebral ventricles [2–4]. The formation of CSF is assumed to involve co-transport of water by proteins located at CP [5–7]. Water is thought to be transported against the osmotic gradient by proteins co-transporting Cl^−^, Na^+^ and HCO_3_^−^ [3, 8]. This is suggested to be mediated by ATP-dependent transporters and ionic gradients, which is highly dependent on the Na^+^/K^+^-ATPase (NKA), Na^+^/K^+^/Cl^−^ cotransporter-1 (NKCC1) and the Na^+^/HCO_3_^−^ antiporter (NBCe2) at the apical membrane facing the ventricles, and possibly also the Na^+^/HCO_3_^−^ cotransporter (NBCn1) at the basolateral membrane [8–11]. Carbonic anhydrases (CA) also play an important role in CSF secretion by catalyzing the generation of HCO_3_^−^ [9, 12]. Over the years it has been demonstrated that cotransporters at CP also contribute to CSF secretion [6, 13–16]. Changing the activity or blocking these transporters and enzymes thus have a great influence on CSF secretion [17].

Although CP is a key structure for maintaining normal brain physiology and may be involved in many CNS disorders such as IIH, Alzheimer’s disorder and multiple sclerosis, the knowledge regarding sex-specificities of CP and how it may be involved in some of the known sex-dependent CNS disorders is widely unknown. The CP is thought to be influenced by circulating sex hormones and a number of studies have shown that CP expresses sex hormone receptors such as androgen receptors (ARs), estrogen receptors (ERs) and progestogen receptors (PR) [18–20]. This localization has been hypothesized to be due to the involvement in regulating the transcriptome of the CP expressing secretory proteins and influence the composition of CSF [21–24]. Sex-related differences in whole transcriptomes from rat CP have also been demonstrated [23]. In contrast, a recent transcriptomic profile study in rats showed a highly shared expression profile between female and male CP [25]. Although, in this study the estrous stage of the female rats was not determined. Interestingly, it has been shown that expression of* Aqp7* and *9* varies during the estrous cycle of mice, suggesting that expression of Aqps may be regulated by various hormonal stimuli and expression patterns is dependent on the estrous cycle stages. However this has not been evaluated in other animal models [26]. Importantly, expression of the AQPs in different tissues have shown to be regulated by hormones [21, 27–29]. It has been shown that gonadectomy in male and females downregulated AQP1 expression in rat nephron and that testosterone was able to upregulate the AQP1 expression strongly [30]. Further, circulating sex hormones may affect CP as it express receptors for these hormones. It has also been shown that gonadectomized female and male rats have profound differences in CP transcriptome compared to sham-operated rats suggesting sex hormones have a central role in CP functions and CSF homeostasis [24]. Therefore, the aim of this study was for the first time to investigate sex-related differences in CP expression of important transporter/channels/enzymes involved in CSF secretion between male and female rats during two different stages of the estrous cycle. In female rats, the estrous cycle has four phases, proestrus (PRO), estrus (ES), metestrus (MET) and diestrus (DI) and lasts for 4 to 5 days. PRO is associated with a rise in circulating estradiol concentrations which leads to increase in lutinizing hormone (LH) and follicle stimulating hormone (FSH) release, similar to the human follicular stage [31]. In ES there is a peak in FSH concentration with an associated rapid decline in estradiol levels corresponding to ovulation. During MET and DI progesterone levels are high, being homologous to human early and late secretory stages of the reproductive cycle [31]. As the serum sex steroid levels varies significantly over the course of the estrous cycle [32], we also aimed to relate the expression of these genes to serum level of steroid hormones in the same rats in order to examine if circulating sex hormones may be related to the transcription of CSF secretory genes at CP.

## Methods

### Animals

20 (10 in MET and 10 in ES) female and 10 male Sprague–Dawley rats (Taconic, Denmark) at the age 10–12 weeks were used, housed in animal facility at Glostrup Research Institute, Denmark. Rats were left 2 weeks to acclimatize prior to all procedures. The rats were fed a standard rodent chow diet (Altromin, Germany) and had access to food and water ad libitum. In the facility, rats were housed at 22 ℃ in a 12:12 h light dark cycle. All experiment were approved by the Danish Animal Experiments Inspectorate (license number 2019-15-0201-00365).

### Vaginal smears- determination of estrous cycle

The estrous cycle stage of each rat was monitored daily by collection of vaginal smears for cytological characterization following a standard protocol [33]. Prior to euthanasia, all rats were allowed to undergo a complete estrus cycle. Euthanasia was performed within a 14-day timeframe, with tissue extraction conducted between 9 and 12 am. Rats in MET and ES were euthanized by 70% CO_2_ followed by decapitation within 30 min after cycle stage determination. ES and MET are easy to distinguish between by the cell type. In ES, cornified epithelial cells are the dominant cell type where MET is characterized by an even distribution of leucocytes, cornified epithelial cells and nucleated cells (see Additional file 1: Figure S2). The rational for choosing ES was that the hormonal concentrations are high in proestrus (the phase just before estrus). Since there is a delay in transcription of genes and proestrus is relatively short (~ 14 h) we would expect to see a difference in the two estrous stages despite the hormonal concentration of progesterone and estradiol are relatively similar (lower concentration in estrus and rising concentrations in metestrus).

### Tissue and serum collection

After the rats were decapitated under CO_2_ sedation, brains were removed quickly and placed in ice cold PBS. The CP from the lateral and fourth ventricles were dissected from the brain and snap frozen on dry ice. The tissue samples were stored at – 80 ℃ until used for subsequent analysis. The blood was collected following decapitation. Whole blood was allowed to clot by leaving it at room temperature for 20 min followed by centrifugation at 2000 × *g* for 10 min at 4 ℃. The supernatant was then immediately transferred and the serum samples were stored at – 80 ℃ until later analysis.

### mRNA expression

The CP were used for mRNA expression experiments. Change in gene expression of *Aqp1* and 4, *Slc12a2* (NKCC1), *Slc4a10* (NCBE), *Slc4a5* (NBCe2), *Car2* (CaII) and *Car3* (CaIII) and subunits of the Na^+^/K^+^-ATPase (Atp1a1, Atp1b1, Fxyd1) were compared between male and female during two different estrous phases, MET and ES. Trizol^™^ was used for isolation of total RNA. Each sample was homogenized in 1 mL TRIzol (Invitrogen^™^, Waltham, MA, USA), whereafter RNA was precipitated in isopropanol. The purified RNA samples were diluted in nuclease-free water (QIAGEN) and afterwards concentrations and quality were measured by UV spectrophotometry on NanoDrop (Thermo Scientific). RNA was converted to cDNA according to the manufacture’s description with Applied Biosystems High-Capacity cDNA Reverse Transcription Kit (Applied Biosystems, Waltham, MA). To access gene expression each sample was ran in single-plex 9 ng cDNA reactions using the Taqman Gene Expression Master Mix (Applied Biosystems^™^) combined with a TaqMan primer/probe set for each of the following targets (all from Applied Biosystems): *Aqp1* (Rn_00562834_m1), *Aqp4* (Rn_01401327_s1), *Slc12a2* (Rn_00582505_m1), *Slc4a5* (Rn_01420902_m1), *Slc4a10* (Rn_00710136_m1), *Car2* (Rn_01462065_m1), *Car3* (Rn_01461970_m1), *Atp1a1* (Rn_01533986_m1), *Atp1b1* (Rn_00565405_m1), *Fxyd1* (Rn_00581299_m1). The reactions were run on QuantStudio^™^ 6 Pro Real-Time PCR System, 384-well (Applied Biosystems^™^). Each gene was ran in triplicate for each sample. Expression of the target gene was normalized to expression of housekeeping gene *Actb* (Rn_00667869_m1), ΔCT = CT (target gene) − CT (reference gene). ΔCT was converted into arbitrary units (AU), $AU={2}^{-\Delta Ct}$.

### Western blot

Protein was obtained by the Trizol reagent (Invitrogen) method after RNA extraction in each sample. Protein was extracted, precipitated and washed accordingly to the manufactures protocol (BioRad, Hercules, CA, USA). The protein was solubilized in lysis buffer with 1% sodium dodecyl sulfate. β-mercaptoethanol was added as a reducing agent making up 5% of the solution. Protein concentration was determined in triplicates on NanoDrop Spectrophotometer (NanoDrop 2000c, Thermo Scientific, Waltham, MA). 25 µg protein was diluted and mixed with LDS sample buffer (Thermo Fisher scientific) and sample reducing agent (Thermo Fisher scientific) followed by separation on a bolt 4–12% bis–tris plus gel (Invitrogen) under gel electrophoresis. Proteins were transferred to a polyvinylidene difluoride membrane by an iblot machine (Invitrogen). The membranes were blocked with blocking buffer (5% non-fat milk in Tris-buffered saline 0,1% Tween 20 (TBST)) and incubated overnight with primary antibodies; 1:1000 Aqp1 (ab168387, Abcam, Cambridge, UK), 1:1000 Car2 (ab124687), 1:500 Atp1a1 (3060S, cell signalling), 1:500 Lamin-B1 (ab65986, Abcam). The membranes were washed with TBST and incubated with secondary antibody 1:10,000 (goat-anti-rabbit (P0448). Bands were detected using chemiluminescent (Amersham ECL, GE Healthcare, Chicago, IL). The reaction was captured on luminescent image analyzer (LAS-4000 Luminescent Image Analyzer, Fujifilm, Tokyo, Japan). Protein bands were quantified using ImageJ (NIH, USA). Gels and samples were ran in parallel and membrane cut according to molecular weight prior to incubation with antibodies. Protein was normalized to the male level, set as 1. Due to protein limit or lack of suitable commercial antibodies, the protein expression of NKCC1 could not be determined. Moreover, numbers are uneven across groups due to protein availability. Raw and annotated blots can be found in Additional file 1: Figure S3.

### Liguid chromatography—mass spectrometry

Prior to quantification of steroid hormones, purification and extraction were performed using solid phase extraction (SPE) followed by LC–MS/MS analysis. A modified version of the method described by Weisser et al. was applied [34]. The data processing from the LC–MS/MS analysis were carried out in MultiQuant v 3.0 software (AB SCIEX). The chromatographic peak was manually integrated for each steroid. Individual datapoints were excluded if the chromatographic peaks were of bad quality or if the peaks were below limit of detection and limit of quantitation. Calculations and graphs were conducted using Microsoft excel 2010 and GraphPad.

### Statistics

The relative gene expression (AU) and correlation to serum hormone level was analyzed using GraphPad Prism (V9.1, Graphpad Software Inc, San Diego, CA). The normality of the data was assessed by Shapiro–Wilk normality test where p < 0.05 was considered significant. For normally distributed genes we did a one-way ANOVA with multiple comparisons (Tukey’s multiple comparisons test). Here we compared both Male vs. MET, Male vs. ES and MET vs. ES. We used Brown-Forsyth and Welch ANOVA where the SD were significantly different: Test performed on Car2, Aqp1, ATP1a1, ATP1b1. Non-normal distributed data differences in gene expression were assessed by Kruskal–Wallis test combined with a post hoc Dunn’s multiple comparisons test: test performed on Slc4a, Car3, FXYd1.

Pearson’s r was used for the correlation analysis for parametric data and Spearmans r was used for non-parametric data. Gene expression data is presented as mean ± SD and correlation data is presented as p-value (p), correlation coefficient (r). Genes that were significantly different expressed were correlated to all the analyzed steroid hormones in the serum. We did not correct for multiple comparisons for the correlation analysis because this would have increased the likelihood of type II statistical errors.

## Results

### Gene expression at CP

#### Males compared to females

**Fig. 1:**
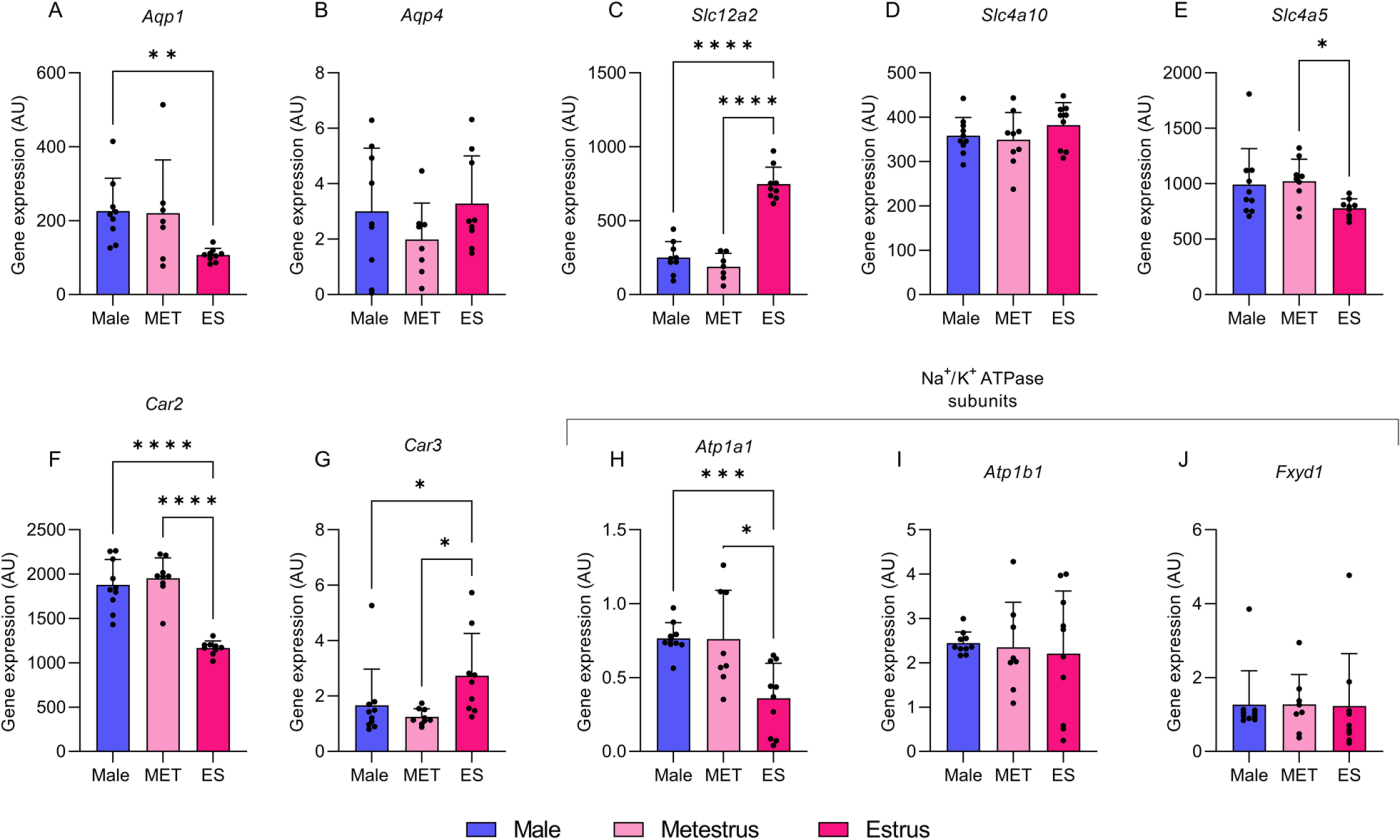
Gene expression at choroid plexus. Gene expression in rat CP from males, metestrus (MET) females and estrus (ES) females. The gene expression includes, *Aqp1, Aqp4, Slc4a5, Slc4a10, Slc12a2, Car2 and Car3, Atp1a1, Atp1b1*, *Fxyd1* and was determined by RT-qPCR. The data is presented as AU, mean ± SD. For **A**, **H** and **I** Brown-Forsyth and Welch ANOVA with post-hoc Dunnets were performed. For **B**, **C**, **D** and** F** One-way ANOVA awith post-hoc Tukey’s test were performed. For **E**, **G** and** J** Kruskal–Wallis test with post-hoc Dunnet’s was performed. The statistics were done on the AU values. *p < 0.05, **p < 0.01, ***p < 0.001, ****p < 0.0001 (n = 8–10)

We found significant changes in gene expression at CP when comparing males to females in ES. Specifically, we found that females in ES had lower *Aqp1* expression by 0.47-fold (226 ± 29 vs 106 ± 6 AU, p = 0.03, Fig. 1A) compared to males. There was no difference in expression of *Aqp4* between the groups (Fig. 1B). Further, a pronounced difference was observed in expression of *Slc2a12* (NKCC1), where females in ES had higher expression by threefold (250 ± 36 vs 747 ± 38 AU, p < 0.0001, Fig. 1C) compared to males. No differences were found between male and females in ES in expression of *Slc4a10* (NCBE) and *Slc4a5* (NBCe2) (Fig. 1D, E). The carbonic anhydrase isozyme *Car2* (CaII) expression was also different when comparing males to ES females where *Car2* was lower by 0.6-fold (1879 ± 90 vs 1167 ± 26 AU, p < 0.0001, Fig. 1F). In contrast, expression of *Car3* was higher by 1.6-fold in ES females (1.7 ± 0.4 vs 2.7 ± 0.5 AU, p = 0.04, Fig. 1G). The catalytic subunit of NKA, *ATP1a1*, showed lower expression in ES females compared to males by 0.5-fold (0.76 ± 0.03 vs 0.36 ± 0.07 AU, p = 0.0008, Fig. 1H). No change was observed in expression of *ATP1b1* or *FXYD1* (Fig. 1I, J). No differences were found when comparing gene expression of the markers between male and female rats during MET.

#### MET females compared to ES females

When comparing females in MET to females in ES, expression of *Aqp1* was lower in ES females, although not significant (221 ± 54 vs 106 ± 17 AU, p = 0.06, Fig. 1A). No difference was found in expression of *Aqp4* (Fig. 1B). A profound difference was found in expression of *Slc2a12* (NKCC1), between MET females and ES females by 4.5-fold (189 ± 34 vs 747 ± 38 AU, p < 0.0001, Fig. 1C). Further, difference in one of two bicarbonate transporters was found when comparing ES female and MET females. ES females had a 0.8-fold lower expression of *Slc4a5* (NBCe2) (1021 ± 200 vs 778 ± 84, AU, p = 0.04, Fig. 1E). Further, both CA isozymes were different when comparing MET females to ES females where *Car2* was lower by 0.6-fold (1954 ± 76 vs 1167 ± 26 AU p ≤ 0.0001, Fig. 1F) while *Car3* was higher by 2.2-fold during ES (1.3 ± 0.1 vs 2.7 ± 0.5 AU, p = 0.03, Fig. 1G). The $\alpha$-subunit of the NKA*, ATP1a1*, showed lower expression in ES females compared to MET females by 0.5-fold (0.76 ± 0.12 vs 0.36 ± 0.075 AU, p = 0.04, Fig. 1H).

#### Western blots

**Fig. 2:**
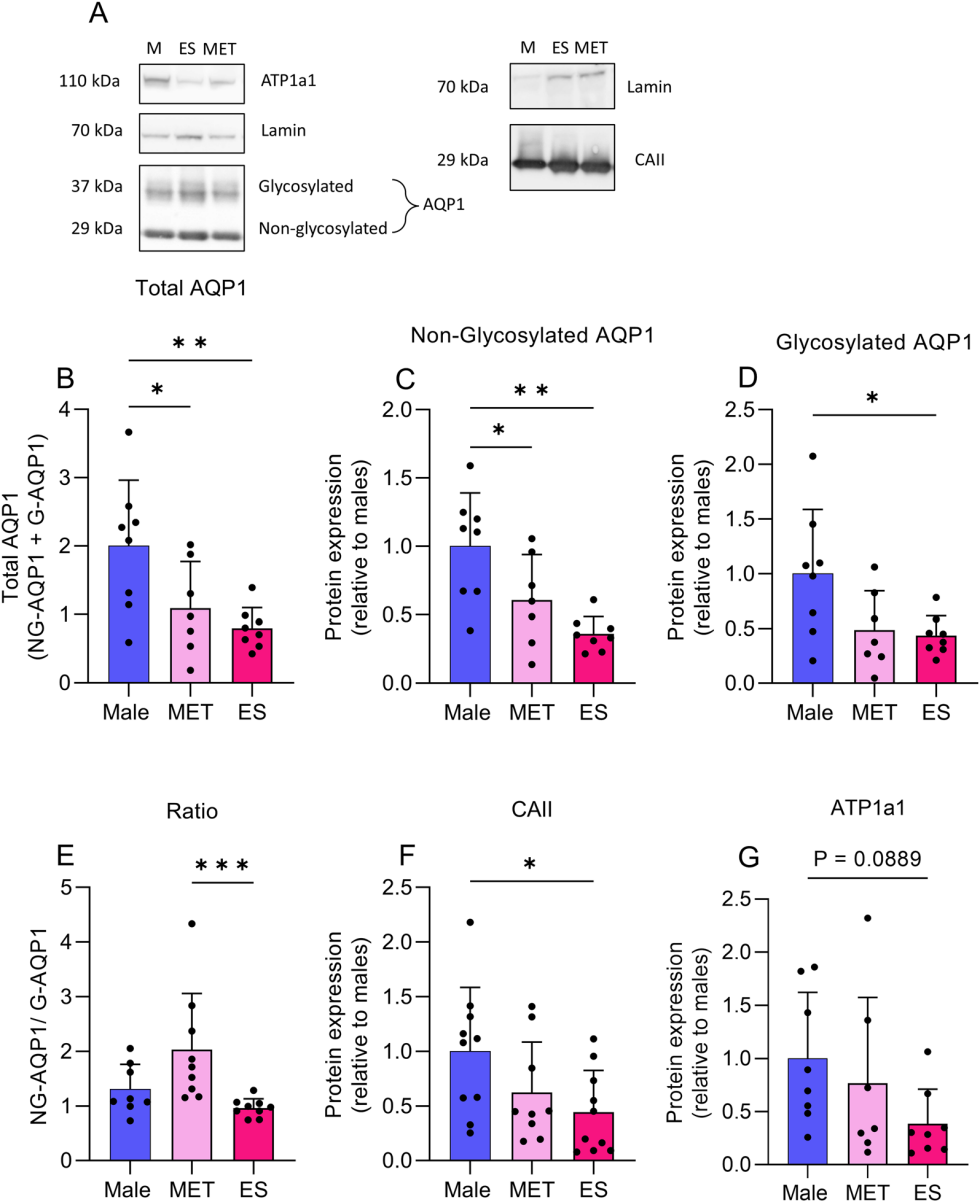
Protein expression at choroid plexus. Western blots on rat CP from males, MET and ES females (**A**), where membranes are cut by molecular weight and the resultant images cropped to show representative bands. ATP1a1, lamin and AQP1 columns represent 1 set of gels ran in parallel and the same well number on each membrane. Lamin and CAII represent another set of gels ran in parallel and the same well number on a membrane. Expression of total AQP1 (**B**), non-glycosylated AQP1 (**C**), glycosylated AQP1 (**D**), non-glycosylated/glycosylated ratio (**E**), carbonic anhydrase 2 (CAII) (**F**) and ATP1a1 (**G**). One-way ANOVA with post-hoc Holm-Sidak’s test for **B**, **C** and **D**. Kruskal–Wallis test with post-hoc Dunn’s test for **E**, **F** and **G**. Data presented as mean ± SD. N = 8–10. *p < 0.05, **p < 0.01 and ***p < 0.001

Given that we identified differences in gene expression at the RT-qPCR level, we performed western blots to identify if the changes translate to the protein level (Fig. 2A). It was found that total AQP1 was decreased in both MET (1.09 ± 0.68, p = 0.043) and ES (0.79 ± 0.30, p = 0.0082) females compared to males (2.00 ± 0.96) (Fig. 2B). We also identified that non-glycosylated AQP1 was reduced in MET (0.60 ± 0.33, p = 0.042) and ES (0.36 ± 0.12, p = 0.0013) females compared to males (1.00 ± 0.39) (Fig. 2C). In contrast, glycosylated AQP1 only decreased in ES (0.43 ± 0.17, p = 0.03) females compared to males (1.00 ± 0.59) (Fig. 2D). MET females (2.03 ± 1.03, p = 0.0007) had an increased non-glycosylated AQP1/glycosylated AQP1 ratio compared to ES females (0.96 ± 0.16) (Fig. 2E). In addition, we found a reduction in CAII expression ES females (0.44 ± 0.38, p = 0.03) compared to males (1.00 ± 0.58) (Fig. 2F). No significant difference in ATP1a1 expression was found. However, there was a trend to decrease of ATP1a1 expression between ES females (0.38 ± 0.32, p = 0.08) and males (1.00 ± 0.62) (Fig. 2G).

## Correlation analysis

### Serum hormone levels and gene expression

**Fig. 3:**
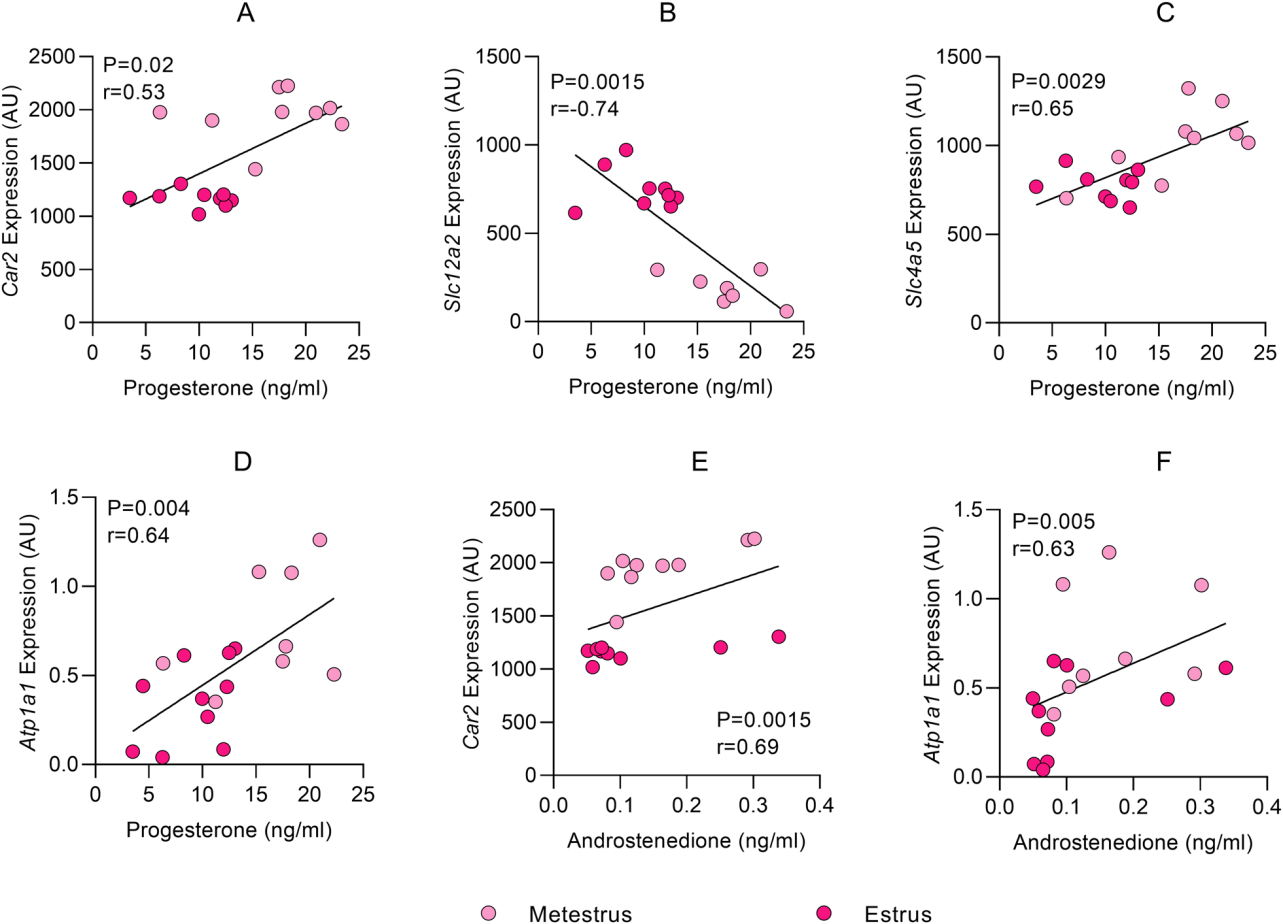
Gene expression in correlation to hormones in the serum. Correlations between steroid hormones in rat serum and gene expression in rat CP. **A** correlation between *Car2* and progesterone, **B** *Slc12a2* and progesterone, **C**: *Slc4a5* and progesterone, **D** *Atp1a1* and progesterone, **E** *Car2* and androstenedione, **F** *Atp1a1* and androstenedione. Spearman’s correlation coefficient for **A**, **B**, **C**, **E** and **F**. **D** was Pearson’s correlations

Gene expression and serum hormone analysis were performed in the same rats thereby allowing for correlation analysis (Fig. 3). The females were analyzed as one group for the correlation analysis. Serum hormone concentrations was assessed for: pregnenolone, progesterone, 11-deoxycorticosterone, corticosterone, 17OH-pregnenolone, 17OH-progesterone, cortisone, dehydroepiandrosterone (DHEA), dihydrotestosterone (DHT), androstenedione, testosterone and 17ß-estradiol (Additional file 1: Figure S1). We performed targeted correlation analysis on those genes that we found statistical difference between to all steroids analyzed (Fig. 1). In female rats gene expression of *Car2* was positively correlated to serum level of progesterone (p = 0.02, r = 0.53, Fig. 3A) and the expression was also positively correlated to serum level of androstenedione (p = 0.0015, r = 0.69, Fig. 3E). Gene expression of *Slc12a2* (NKCC1) showed a moderate negative correlation with serum level of progesterone (p = 0.015, r = -0.58, Fig. 3B). Further, gene expression of *Slc4a5* (NBCe2) was positively correlated to serum level of progesterone (p = 0.001, r = 0.69, Fig. 3C). Lastly, *Atp1a1* was positively correlated to serum level of progesterone (p = 0.004, r = 0.64, Fig. 3D) and also positively correlated to the serum level of androstenedione in female rats (p = 0.005, r = 0.63, Fig. 3F). We found no significance with the other steroids assessed.

## Discussion

Little is known about sex-specificities of CP which is the main tissue producing CSF. Therefore, this study aimed at resolving sex-differences in the expression of important choroidal transport proteins. The current study demonstrated that there are sex-dependent differences in expression at CP in important transporters, channels and enzymes involved in CSF secretion when comparing male rats to age matched female rats during two different estrous cycle stages. Here we showed for the first-time significant differences in expression of *Aqp1*, *Slc12a2* (NKCC1), *Car2*, *Car3* and $\alpha$-subunit of NKA, *Atp1a1*, when comparing male rats to females in ES. The gene expression data of AQP1 and CAII was also confirmed by protein data. All these choroidal proteins are implicated in CSF secretion and highly expressed at CP. Further, we found that gene expression of some of CSF proteins investigated in this study were correlated to serum levels of the steroids progesterone or androstenedione in the female rats, suggesting that hormonal levels may influence CP function and dynamics.

A growing body of research has shown sex differences in the prevalence and symptomatology of many CNS disorders including CSF pathologies such as IIH. Although IIH is overrepresented in women, it has not been fully determined, whether IIH patients have increased CSF secretion, reduced CSF drainage or a combination of those. However, as IIH patients are mainly females of childbearing age, it is possible that steroid hormones have a pathogenic role in IIH. Therefore, it is of great importance to identify sex-related differences in order to understand how this may contribute to disease presentation. In this study we selected to examine the gene expression in female rats during MET and ES. A limitation of the study is that pituitary drivers of sex hormones, LH or FSH were not assessed. We found no differences in gene expression between female rats in MET and males. Interestingly, extensive differences in gene expression were found in ES female rats both compared to male but also MET females. In the expression of water channels involved in CSF secretion, we found lower expression of Aqp1 but not of Aqp4 in females during ES compared to males. The difference in gene expression of Aqp1 was confirmed by protein expression where the protein level of AQP1 was lower in female ES compared to male. In support, it has been shown that there is gender specific expression of AQP1 in the rat nephron, where AQP1 protein and mRNA expression was higher in adult males compared to females [30]. We also compared ES females to MET females, where ES females had a tendency of lower AQP1 in gene expression. Further, in another study it has been shown that expression and staining pattern of AQP7 and 9 are different during stages of mouse estrus cycle which emphasizes the importance of taken the estrous cycle in account as performed in the current study [25]. In contrast to these results, no differences in expression of AQP1, 2 and 4 in mice CP were identified between male and females in different stages of the estrous cycle [35]. However, the limitation of that study was that the protein levels were only evaluated by immunohistochemistry, which is not an optimal quantification method.

Over the recent years is has been demonstrated that co-transporters such as NKCC1 at CP contribute more to CSF secretion than the AQPs [13, 14]. Here we describe differences in gene expression in some of these important ion-transporters involved in the CSF formation in female rats during ES compared to male and female rats in MET. NKCC1 is robustly expressed at the CP and localized in the luminal membrane facing the ventricles, where it is thought to have an outward directed transport, and therefore acts as a key contributor to CSF secretion, as previously described in mice, rats and dogs [5, 17, 36, 37]. We found that NKCC1 displayed the highest degree of change by threefold increase in ES females compared to males. This suggests that female rats may have increased CSF secretion during ES however more research is needed to explore this. The observed differences of NKCC1 in this study needs to be evaluated by protein level and protein activity in future studies. For the other exchangers, we found that expression of NCBE was not different in any of the groups while the expression of NBCe2 was lower in ES females compared to MET females. Further, in expression of the three subunits of NKA we found that Atp1a1 expression was lower in ES females compared to males and MET females. The effect of the observed changes of the CP transporters needs to be further examined in order to understand how this will impact CSF and ICP dynamics. CA is an enzyme highly expressed at CP and involved in the regulation of CSF secretion by indirectly reducing cerebral water transport. Therefore, CA inhibitors such as acetazolamide are used as first line pharmacological treatment for elevated ICP [38]. Here we demonstrated that gene and protein expression of *Car2* was profoundly decreased while gene expression of *Car3* was higher in ES compared to male and females in MET. It remains to be determined whether the expression of these transport proteins with different expression levels may be functionally different and physiologically relevant during disease stage. Further, the protein data revealed no differences in expression of these proteins between ES and MET females as observed in the gene expression data. This may be due difference cycle lengths (ES 24–48 h, MET 6-8 h) and the latency for protein turnover.

Altogether, the current work shows clear sex-differences in expression of choroidal proteins involved in CSF secretion dependent on the estrous cycle stage. Sex-related specificities of CP has been suggested by cDNA microarrays studies with differences in CP transcriptome between female and male rats in pathways related to circadian rhythm signalling, metabolism, neurogenesis and stem cell differentiation [23]. In support, sex-related differences in CSF protein composition have been shown [24]. However, a more recent transcriptomic study did not show any sex-related differences in all genes or for transporters/channels involved in CSF secretion [25] although with the limitation that the estrous cycle of the female rats were not determined which may explain the high overlay of genes in female and male rats. Further, gene expression may not mirror the quantitative expression at protein level or protein activity and a limitation with these studies is that protein expression was not assessed. Nevertheless, in the current study, we demonstrated that there are sex-differences in expression of these transporters when taking the estrous cycle in account. This has been lacking in previous studies which demonstrated RNA transcripts at CP. Although the current study demonstrated profound differences of several membrane transport proteins suggested to be involved in CSF secretion, their individual function or net effect on CSF secretion levels were not assessed. Our data reveals differences in various ion transporters and exchangers in the CP during the female cycle, however the impact on the CSF secretion rate cannot be determined. Thereby, it can only be speculated that female rats may have changed CSF secretion during ES however more research is needed to explore this. In support of this theory, a recent study demonstrated the CSF production in young female mice was 30% higher compared to young male mice [39]. Further, obese female rats have also been found to present with elevated CSF secretion compared to obese male rats [40]. Taken these data together with our study, we suggest that expression of certain CSF secretion transporters at CP is sex and cycle-dependent and may thereby affect CSF homeostasis. Future work on assessing the role of how CSF and ICP may vary with the female cycle is warranted.

It is not known how the expression of the sex steroid receptors vary with the cycle. In order to understand if circulating sex hormones may be correlated to the gene expression at CP, we measured the serum level of steroid hormones in the same rats by LC–MS/MS. By this we found that expression of *Car2* was positively correlated to both progesterone and androstenedione levels. Androstenedione is an androgen, but with a much lower affinity for the androgen receptor compared to testosterone. However, androstenedione can easily and rapidly be transformed into testosterone in a single enzymatic step by the 17β-hydroxysteroid dehydrogenase, which the female rat CP expresses [41]. Expression of NKCC1 in female rats during MET and ES was moderately inversely correlated to serum level of progesterone. Little is known about hormonal regulation of NKCC1 expression or activity. To our knowledge the correlation between progesterone and NKCC1 has not been showed before and the hormonal regulation on expression and activity is thereby unknown. Further, we found that progesterone also positively correlated to gene expression of NBCe2 and Atp1a1. One older study showed that exposing rabbit CP to progesterone in combination with 17-β-estradiol reduced the activity of Na^+^/K^+^-ATPase and other ATPases [42]. The subunit Atp1a1 also positively correlated to the serum level of androstenedione. Interestingly, testosterone has been associated with an increased activity of NKA in cultured CP cells which associated with increased CAII and CAIII expression [43]. The data in the present study is based on correlation analysis and therefore the direct evidence of change in mRNA expression at CP cannot be concluded. Although this study supports the hypothesis that level of sex hormones may impact expression of various transporters at CP, futures studies investigating the direct impact of each hormone needs to be performed.

Although this works focus was on sex steroids, other steroids namely the corticosteroids have their receptors expressed at the CP [44, 45]. To assess the effects of glucocorticoids and mineralocorticoids and their cognate receptors on the expression of genes at the CP, future chronotyping studies will be required due to the diurnal variation of corticosteroids.

## Conclusions

Sex-related differences in brain pathophysiology involving CSF and CP still pose many unanswered questions complicating the understanding of pathophysiology and the treatment strategies. The current study demonstrated profound sex-related differences in expression of key transporters/channels/enzymes involved in CSF secretion that were dependent on the estrous cycle. This study proposes for a hormonal regulation of the expression of CSF transporters which may have great importance in the understanding of CSF disorders. Further, the study suggests that future studies on CP physiology and related pathology should take the cycle determination of female rats in consideration.

### Supplementary Information

**Additional file 1: Figure S1.** Steroid profile of serum in males and females in metestrus and estrus as measured by LCMS.**Figure S2.** Representative light micrographs of vaginal smears in each phase in the estrus cycle of female rats. **Figure S3.** Raw and annotated western blot images for lamin-B1, AQP1, ATP1a1 and CAII.

## Data Availability

All data generated or analysed during this study are included in this published article [and its Additional files].

## Abbreviations

CA: Carbonic anhydrases
CSF: Cerebrospinal fluid
CP: Choroid plexus
CPe: CP epithelial
ES: Estrous
FSH: Follicle stimulating hormone
IIH: Idiopathic intracranial hypertension
ICP: Intracranial pressure
LH: Lutinizing hormone
MET: Metestrus
NKA: Na^+^/K^+^-ATPase
NKCC1: Na^+^/K^+^/Cl^−^ cotransporter-1
NBCe2: Na^+^/HCO3^−^ cotransporter
NBCn1: Na^+^/HCO3^−^ cotransporter
