# Supplementary material for: Cycle-dependent sex differences in expression of membrane proteins involved in cerebrospinal fluid secretion at rat choroid plexus
Source: BMC Neurosci. 2023 Nov 9;24:60. doi: 10.1186/s12868-023-00829-w (PMC10633912; doi:10.1186/s12868-023-00829-w)
Supplement: Supplementary file 1 — Additional file 1: Figure S1. Steroid profile of serum in males and females in metestrus and estrus as measured by LCMS.Figure S2. Representative light micrographs of vaginal smears in each phase in the estrus cycle of female rats. Figure S3. Raw and annotated western blot images for lamin-B1, AQP1, ATP1a1 and CAII. [file 12868_2023_829_MOESM1_ESM.docx]

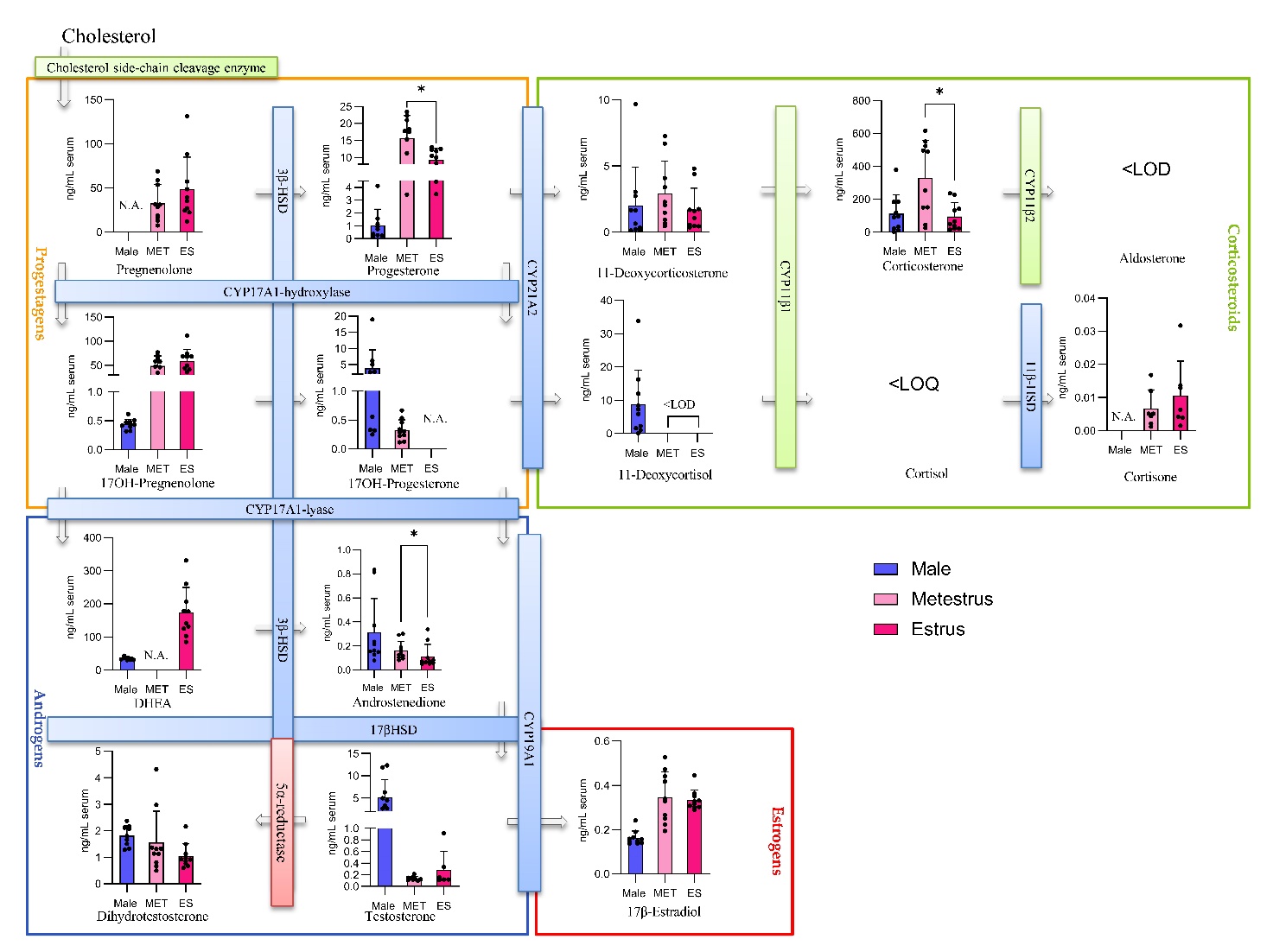


**Figure S1: Steroid profile in serum of male, metestrus female and estrus female rats:**

The figure includes concentrations of steroid hormones and their intermediates as well as converting enzymes. Steroids produced by the adrenal cortex, ovaries, and testis are outlined in boxes of green, red, and blue, respectively and progestogens are outlined in orange. Indicating the major enzymes CYP450 and HSD marked in blue or green referring to their cellular location. Blue is located in the endoplasmic reticulum and green is located in the mitochondria. The concentrations in male (n=9-10), metestrus female (n=7-10) and estrous female (n=6-10). The data is presented in ng/mL serum. Bad quality: Progesterone Male 1 exclude, Dihydrotestosterone male 5 exclude, Pregnenolone all male excluded, cortisol all male excluded. Cortisone female ES 2,6,8 excluded, Testosterone female ES 2, 6, 7, 10, 17OH-progesterone all female ES. Corticosterone, female MET 3,7,10 exclude, testosterone female MET 7 exclude, DHEA all female MET excluded. LOD: 11-deoxycortisol all females ES excluded, 11-deoxycortisol all females MET excluded. LOQ: cortisol all female ES excluded, cortisol all females MET excluded. <LOD: the concentration was below the limit of detection. N.A: the data it could not be analyzed accurately and were excluded, because the peaks were broad. Blue: male, light red: metestrus female, dark red: female estrous.


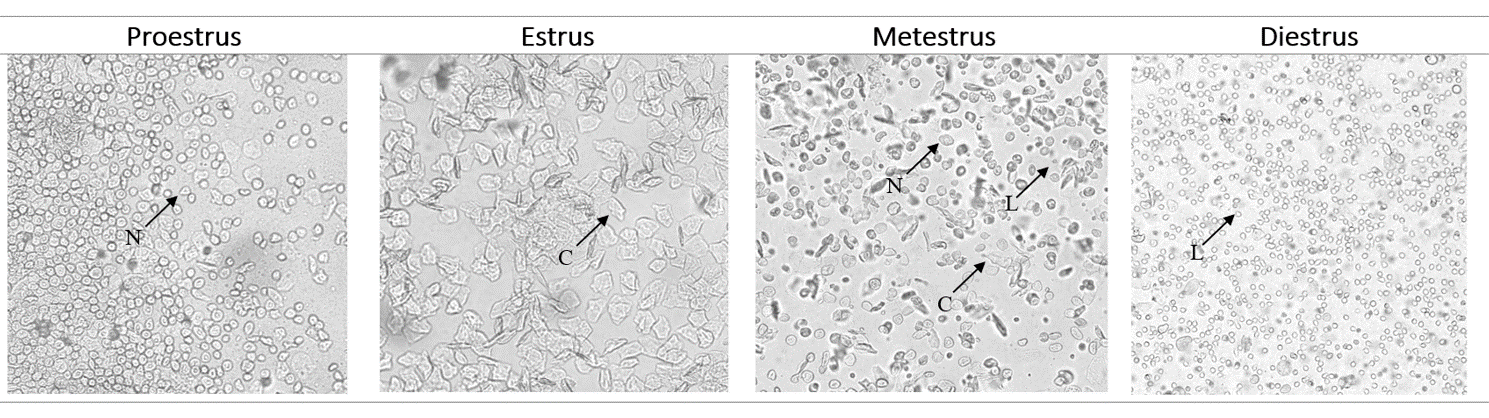


**Figure S2:** **Microscopic vaginal smear pictures:** The three cell types that appear during the estrous cycle are nucleated epithelial cells, cornified epithelial cells and leucocytes, and the cycle stage can be estimated by examining the proportion of cells in vaginal secretion through a vaginal smear. The different phases of the estrous cycle (Proestrus, estrus, diestrus and metestrus) determined by the distribution of different cells. The cell types are specified as N = nucleated, L = leucocytes and C= cornified.


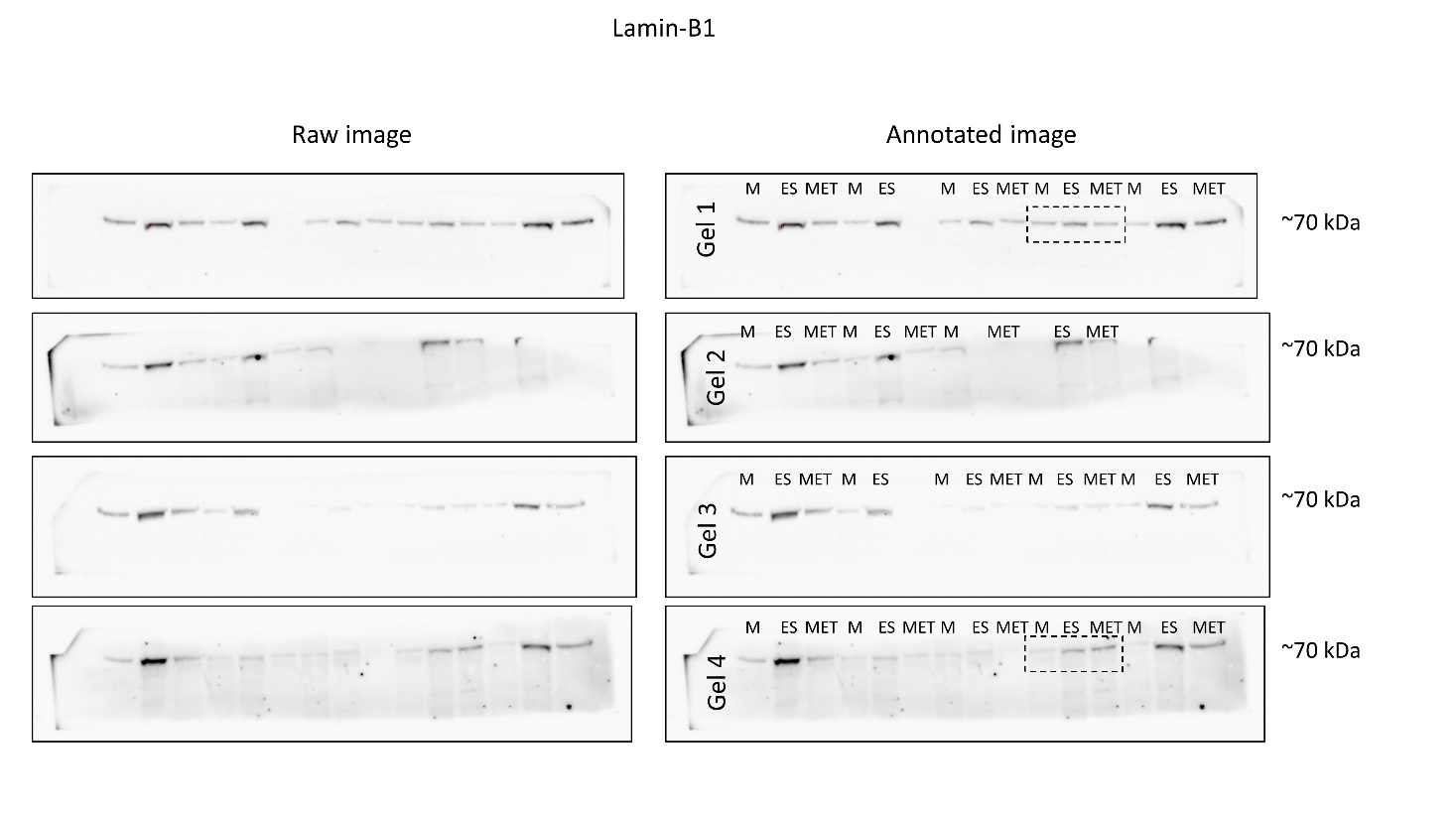


**Figure: S3:**

Raw lamin-B1 immunoblots

Lamin immunoblots, where membranes were cut according to molecular weight prior to immunoblotting. For the manuscript the membranes were cropped to show representative bands as denoted by the dotted inset lines. M= male, ES= female estrus and MET= female metestrus. The image also contains the molecular weights.


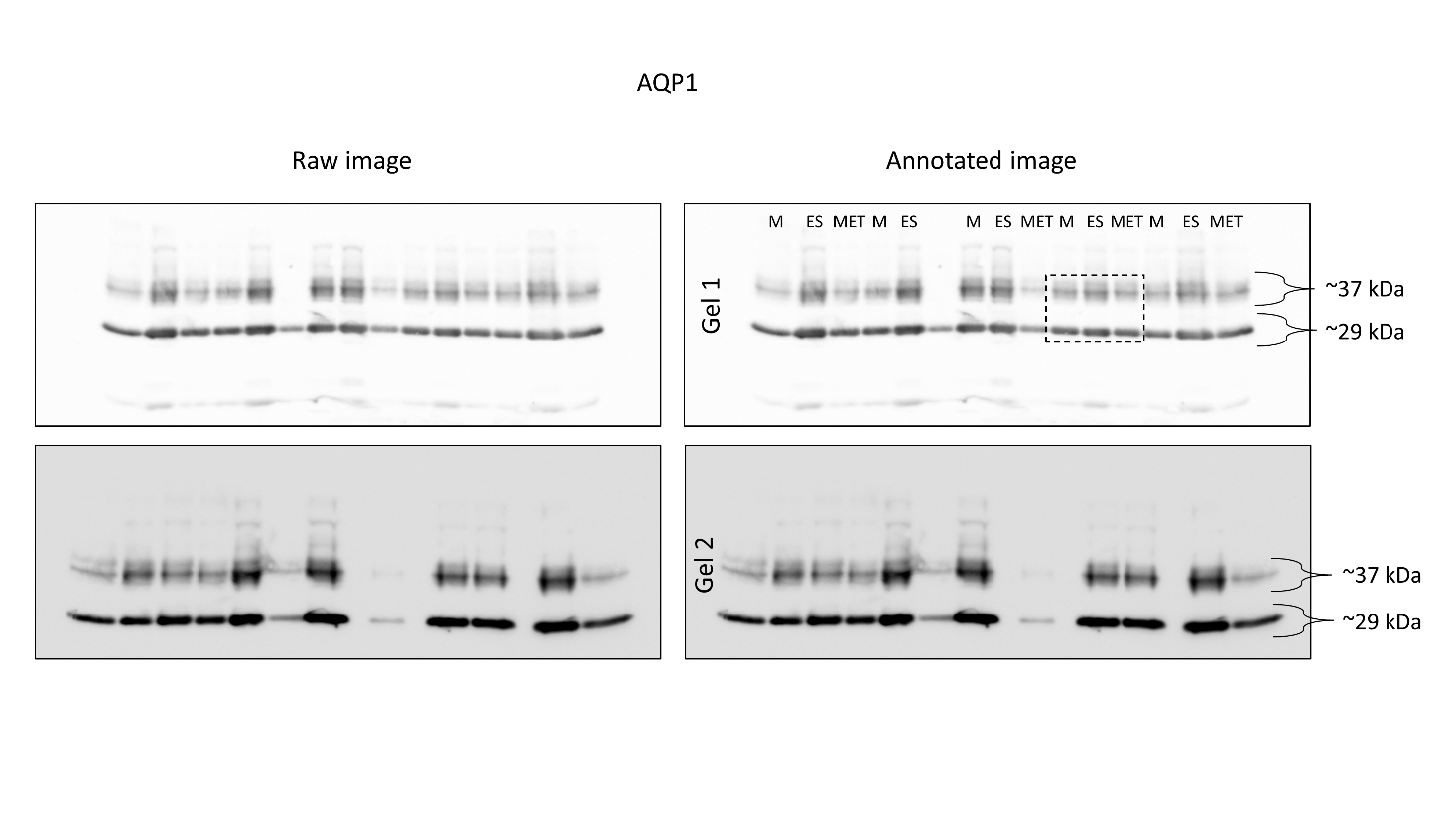


Raw AQP1 immunoblots

AQP1 immunoblots, where membranes were cut according to molecular weight prior to immunoblotting. For the manuscript the membranes were cropped to show representative bands as denoted by the dotted inset lines. M= male, ES= female estrus and MET= female metestrus. The image also contains the molecular weights.


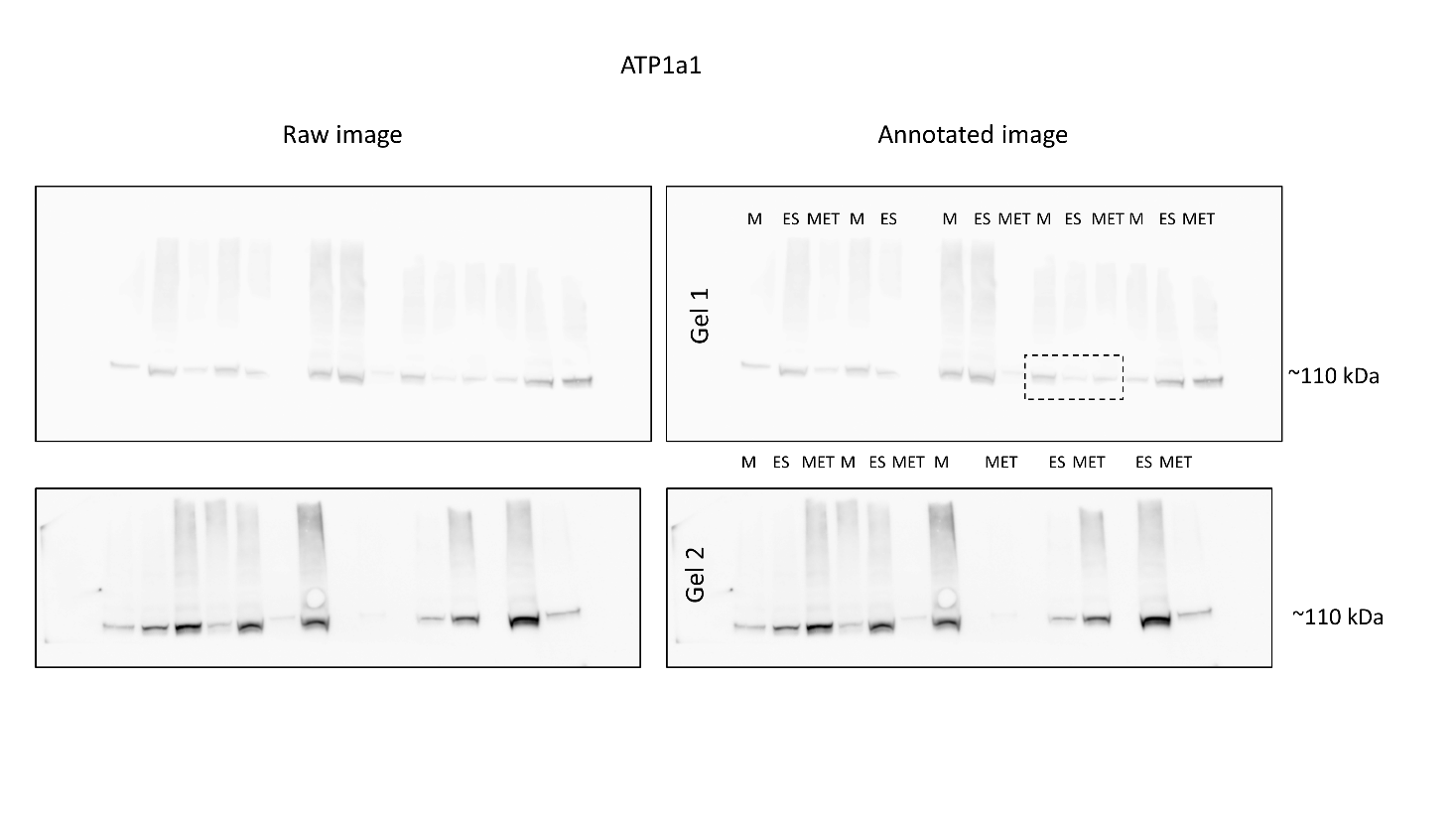


Raw ATP1a1 immunoblots

ATP1a1 immunoblots, where membranes were cut according to molecular weight prior to immunoblotting. For the manuscript the membranes were cropped to show representative bands as denoted by the dotted inset lines. M= male, ES= female estrus and MET= female metestrus. The image also contains the molecular weights.


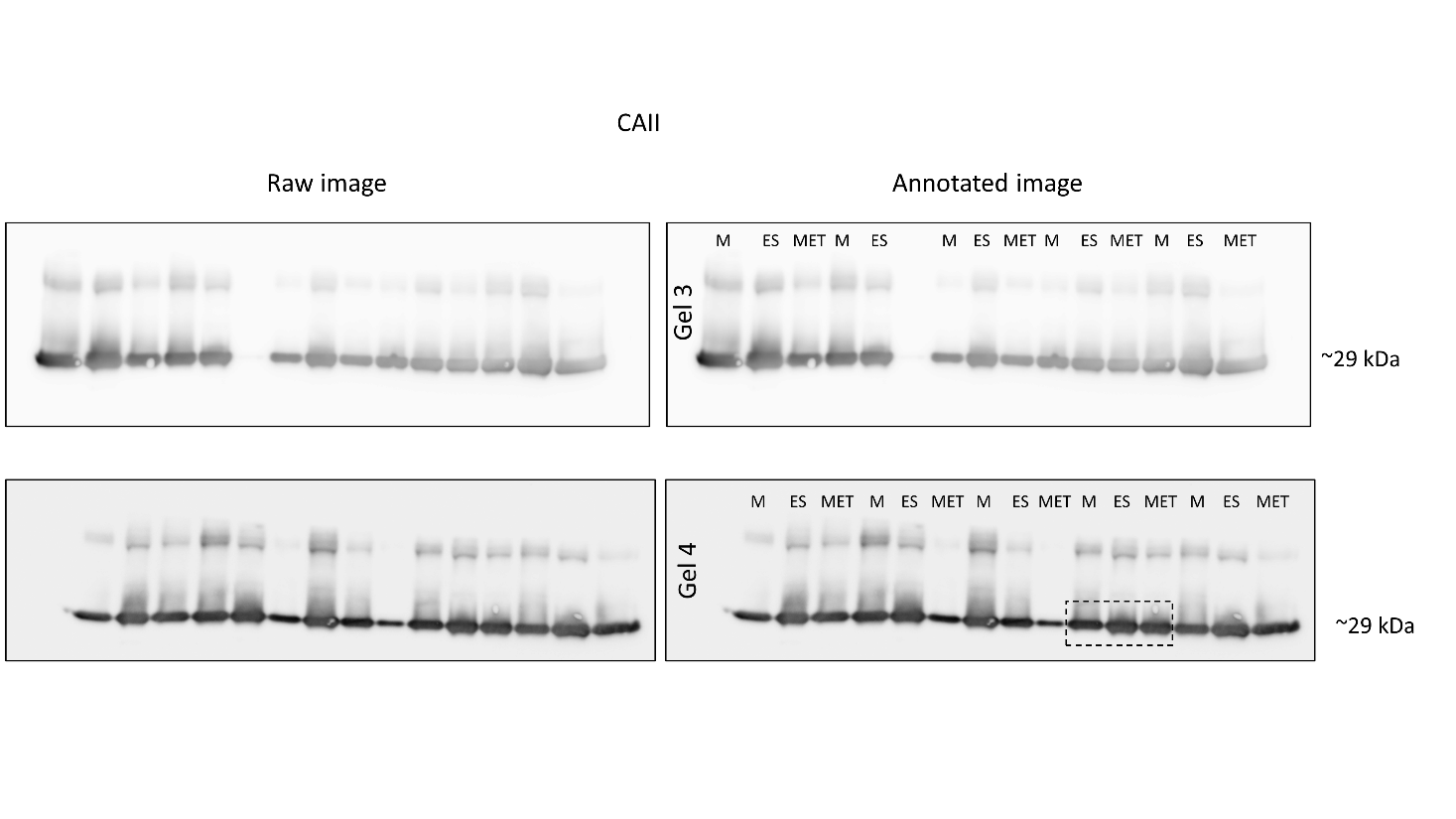


Raw CAII immunoblots

CAII immunoblots, where membranes were cut according to molecular weight prior to immunoblotting. For the manuscript the membranes were cropped to show representative bands as denoted by the dotted inset lines. M= male, ES= female estrus and MET= female metestrus. The image also contains the molecular weights.
